# Gene cassettes of class I integron-associated with antimicrobial resistance in isolates of *Citrobacter spp.* with multidrug resistance

**Published:** 2018-02

**Authors:** Roya Chegene Lorestani, Alisha Akya, Azam Elahi, Yazdan Hamzavi

**Affiliations:** 1 Nosocomial Infection Research Center, Faculty of Medicine, Kermanshah University of Medical Sciences, Kermanshah, Iran; 2 Department of Parasitology and Mycology, Kermanshah University of Medical Sciences, Kermanshah, Iran

**Keywords:** *Citrobacter*, Gene cassettes, Integrons, Multidrug-resistant

## Abstract

**Background and Objectives:**

Integrons play a major role in the transmission and accumulation of resistance factors in multidrug resistant bacteria. This study was aimed to evaluate the gene cassettes of class I integron and antimicrobial resistance in isolates of *Citrobacter* with multidrug resistance (MDR).

**Materials and Methods:**

Ninety isolates of *Citrobacter spp.* were collected from the largest hospital in Kermanshah, Iran. Antimicrobial resistance patterns were determined using disc diffusion method. The class I integron were detected by PCR. The integrase positive isolates were further analyzed for the presence of gene cassettes using 5′ and 3′ conserved sequences (CSs) primers and PCR products were sequenced. The data were analyzed using the chi-square test.

**Results:**

Of 90 *Citrobacter* isolates, 46 (51.1%) were multidrug resistant. Class I integron and gene cassettes were determined in 30 isolates (65.2%). Gene cassettes were found which contained genes encoded resistance to aminoglycosides and trimethoprim and a putative gene. Gene cassettes of *dfrA12-orfF-aadA2, dfrA1-aadA1, aadA1* and *dfrA15-aadA2* were also found in *Citrobacter* isolates.

**Conclusion:**

Our results indicate there is a high frequency of class I integron among multi-drug resistant strains of *Citrobacter* isolated from clinical settings. A high frequency of class I integron associated gene cassettes, in particular *dfr* and *aadA,* present in MDR strains of *Citrobacter*. This data indicates an important role of integrons in the creation and transmission of MDR strains in health care centers.

## INTRODUCTION

*Citrobacter* species have been reported as nosocomial pathogens with multidrug resistance (MDR) in many countries since recent decades (1). *Citrobacter* species have involved in various human infections, in particular, urinary tract infections, wound infections and respiratory infections (2). The antibiotic resistance of this group of bacteria have increased and MDR isolates have frequently been reported (2). The horizontal gene transferring plays the main role in the spread of antibiotic resistance genes and subsequently the rapid emergence of antibiotic resistance among *Enterobacteriaceae* (3). The mobile genetic elements such as plasmids, transposons and integrons are main factor for horizontal spreading of resistance genes (3). Integrons are conserved DNA sequences, which can efficiently acquire and transfer the resistant genes among bacteria and usually located on mobile genetics elements (4). There are several different classes of integrons, each encodes a distinct integrase gene (4). Class I integron is the most common type presented in clinical isolates of the *Enterobacteriaceae* (5). It is capable to carry single or multiple gene cassettes, which confer resistance to various antibiotics including, aminoglycoside, β-lactams, chloramphenicol, quinolones and trimethoprim (6). Class I integron has two conserved segments; 5′-CS and 3′-CS, separated by a variable region, included the integrated gene cassettes (7). The 5′-CS encodes integrase, located next to the recombination site (*att1*) recognized by the integrase and the promoter (P) which controls the transcription of integrated gene cassettes (7). The 3′-CS usually includes truncated *qac*E (qacED1) and *sul1* genes that confer resistance to quaternary ammonium compounds and sulfonamides, respectively (7). Recombination between the *attl* of integron and *att*C sites of gene cassettes leads to the insertion of gene cassettes downstream to the resident promoter mediated by integrase (7). Integrase is a member of the tyrosine site-specific recombinase family that catalyze the excision and integration of DNA fragments, including gene cassettes (7). Near two hundreds of different cassette arrays have been identified that are flanked by the 5′-CS and 3′-CS endes (6). A strong association of integrons associated gene cassettes with MDR isolates of *Enterobacteriaceae* has been found (8, 9). Gene cassettes encode resistance to various antimicrobial agents, including dihydrofolate reductases (*dfr*), chloramphenicol acetyl-transferases (*cat, cml*), β-lactamases (*bla*), aminoglycoside-modifying enzymes (*aac, aad, aphA*) and ADP-ribosyl transfer-ases (*arr*) have been frequently identified within integrons (8, 9). This study aimed to evaluate the gene cassettes of class I integron-associated antimicrobial resistance in isolates of *Citrobacter* with multidrug resistance (MDR).

## MATERIALS AND METHODS

### Bacterial isolates.

In this descriptive study, 288 different clinical samples (e.g., wound, blood, urine, stool, and other samples) from patients admitted in the largest hospital in Kermanshah were collected during 2014–2015. Using the bacteriological and API20E Kit (bio-Merieux, France) testing, 90 *Citrobacter* isolates were confirmed.

### Antibiotic susceptibility testing.

Antimicrobial susceptibility testing for 16 antibiotics was carried out using the disk diffusion method as recommended by Clinical and Laboratory Standard Institute (CLSI) (10). The antibiotic discs were ampicillin (10μg), cefotaxime (30μg), cefpodoxime (10μg), ceftazidime (30μg), ceftriaxone (30μg), tobramycin (10μg), gentamicin (20μg), ciprofloxacin (5μg), tazobactam/ piperacillin (10μg), cefazolin (30μg), cotrimoxazole (25μg), imipenem (10μg), aztreonam (30μg), ertapenem (10 μg), meropenem (10 μg) and streptomycin (10 μg) (MAST, England). The *Escherichia coli* ATCC 25922 was used as a control. MDR was defined as resistance to at least one antibiotic in three or more classes of antibiotics (6).

### Polymerase chain reaction-detection of class I integron.

The presence of class I integron was screened by PCR using intIF and intIR primers (SinaColon, Iran) (Table 1). Each single reaction mixture (25 μl) contained 2μl of DNA suspension, 10 pmol of each primer, 2x GoTaq Green Master Mixture (SinaColon, Iran). The PCR conditions were as follows; 94°C for 5 minutes, followed by 35 cycles at 94°C for 45 seconds, 55°C for 45 seconds, 72°C for 45 minutes and final extension at 72°C for 5 minutes.

**Table 1.** Oligonucleotide primers used

**Primer**	**Oligoneucleotide sequence (5′-3′)**	**Amplicon size (bp)**	**Reference**
Int1F	CAGTGGACATAAGCCTGTTC	160	11
Int1R	CCCGAGGCATAGACTGTA	160	
5′ -CS	GGCATCCAAGCAGCAAG	variable	11
3′ -CS	AAGCAGACTTGACCTGA	variable	

### Detection of the variable region of class I integrons.

PCR was performed with class I integrase positive isolates using two primers 3′CS and 5′CS (SinaColon, Iran) (Table 1) to amplify the variable region of integron. Each single reaction mixture (25μl) contained 2 μl of DNA suspension, 10 pmol of each primer, 2 × GoTaq Green Master mixtures. PCR reactions began with 5 min of primary denaturation at 94°C followed by 35 cycles of 94°C for 1 min, 58°C for 1 min and finally 72°C for 1 min. The final extension was performed at 72°C for 10 min. After electrophoresis of PCR products on 1% agarose gel (Merck Co, Germany) and staining with ethidium bromide, the gels were visualized by Gel-Documentation apparatus (Bio Rad, USA).

### DNA sequence analysis.

A number of PCR products with sharp bands were cut and purified using the QIA quick PCR purification Kit (QIAGEN, Germany) followed by sequencing. The DNA sequences were performed using an ABI 3730XL DNA analyzer (Macrogen Inc., Korea). Sequences were analyzed using BLAST search (http://www.ncbi.nlm.nih.gov/BLAST).

### Statistical analysis.

Data were recorded and entered into an Excel file. Statistical analyses were performed using SPSS software (Version 20). Variables were analyzed by the Chi-square test. A p-value of < 0.05 was set as the statistical significance of all analyses.

## RESULTS

### Clinical data.

All 90 isolates were from hospitalized patients and confirmed by API20E Kit. They included 77 (85.5%) and 13 (14.4%) *C. freundii* and *C. koseri*, respectively. The clinical samples were included urine (n=46, 51.1%), blood (n=18, 20%), stool (n=11, 12.2%), Respiratory tract secretions (n=11, 12.2%) and wound (n=4, 4.5%). The patients were 49 females (54.4%) and 41 males (45.6%) with the average age of 41.6±26-year-old.

### Antibiotic susceptibility results.

**Fig. 1.**
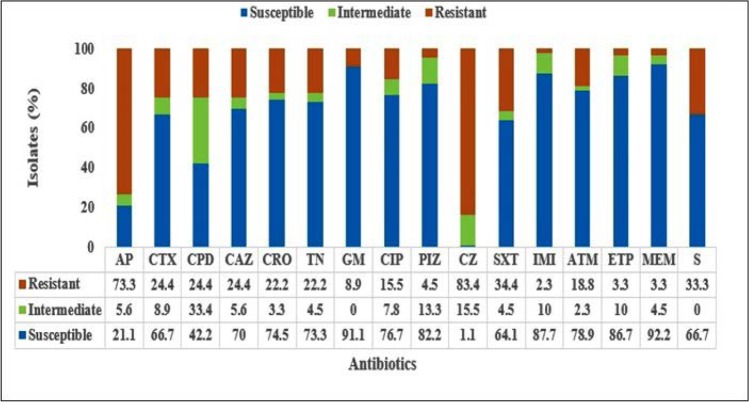
Antimicrobial susceptibility patterns of 90 *Citrobacter* isolates. SXT: Trimethoprim-sulfamethoxazole, CIP: Ciprofloxacin, TN: Tobramycin, GM: Gentamicin, CRO: Ceftriaxone, CTX: Cefotaxime, CAZ: Ceftazidime, ATM: Aztreonam, AP: Ampicilin, S: Streptomycin, CZ: Cefazolin, CPD: Cefpodoxime, ETP: Ertapenem, MEM: Meropenem, PTZ: Tazobactam piperacillin, IMI: Imipeneme.

Results showed high-level of resistance to cefazolin (83.4%) and ampicillin (73.3%). Resistance rates for imipenem (2.3%), ertapenem (3.3%), meropenem (3.3%) and tazobactam/piperacillin (4.5%) was lower (Fig. 1.) Forty six isolates (51.1%) showed MDR phenotype. MDR isolates were mainly isolated from urine (43.5%), blood (21.7%), respiratory tract secretions (17.4%), stool (10.9%) and wound (6.5%).

### Prevalence of class 1 integrons and gene cassettes.

Of the 46 isolates with multidrug resistance, class I integron was detected in 30 (65.2%) isolates. Following PCR amplification of variable region of class 1 integrons, all isolates with class I integron contained gene cassettes with different sizes (500, 600, 700, 750, 1000, 1600 and 1700) (Table 2). The cassettes were in the form of 8 electrophoretic patterns which include three patterns with single band (750, 700, 1600 bp), two patterns with double bands (1600-1000, 1600-1700 bp), one pattern with four bands (1700-1600-1000-750 bp), one pattern with five bands (1700-1600-1000-600-500 bp) and one pattern with six bands (1700-1600-1000-750-700-500 bp) (Table 2). Sequence analysis revealed the different variants of *aadA* (*aadA1, aadA2*) and *dfrA* (*dfrA1, dfrA12, dfrA15*) gene cassettes. The sequence analysis also showed the 1700 bp fragment with 98% homology to *dfrA12-orfF-aadA2* gene, the 1600 bp fragment with 100% identical to *dfrA1-aadA1* gene, the 750 bp gene cassette fragment with 100% identical to *dfrA15-aadA1* gene and the 1000 bp fragment was 99% homology to *aadA1* gene.

**Table 2.** Characterization of Class1 integrons and gene cassettes in 30 MDR isolates of integron-carrying *Citrobacter.*

**Isolates**	**Source**	**Hospital Ward**	**Antibiotic resistance profile**	***intI***	**Size of gene cassettes (bp)**	**Gene Cassettes**
S1	Urine	Surgery	AP/CTX/CPD/CAZ/CRO/CIP/CZ/ATM/S/ SXT	+	750	*dfrA15-aadA1*
S75	Urine	Surgery	AP/CIP/SXT/S	+	750	*dfrA15-aadA1*
S2	Urine	Internal	AP/CTX/CPD/CAZ/CRO/CZ/ATM/S/SXT/TN/GM	+	1600, 1000	*dfrA1-aadA1, aadA1*
S6	Urine	Emergency	AP/CZ/SXT/S	+	1600, 1000	*dfrA1-aadA1, aadA1*
S14	Urine	Internal ICU	AP/CTX/CPD/CAZ/CRO/TN/CIP/CZ/ATM/S/SXT	+	1600, 1000	*dfrA1-aadA1, aadA1*
S17	Urine	Surgery	AP/CAZ/CZ	+	1600, 1000	*dfrA1-aadA1, aadA1*
S26	Urine	Surgery	AP/CTX/CRO/CZ	+	1600, 1000	*dfrA1-aadA1, aadA1*
S84	Respiratory secretions	Infectious	AP/CTX/CPD/CAZ/CRO/PTZ/CZ/SXT/S	+	1600, 1000	*dfrA1-aadA1, aadA1*
S86	Blood	Pediatric	AP/CTX/CZ/S	+	1600, 1000	*dfrA1-aadA1, aadA1*
S94	Stool	Infectious	AP/CTX/CPD/CAZ/ TN/ CZ	+	1600, 1000	*dfrA1-aadA1, aadA1*
S97	Urine	Surgery	AP/TN/CZ	+	1600, 1000	*dfrA1-aadA1, aadA1*
S99	Urine	Pediatric	AP/CZ/S	+	1600, 1000	*dfrA1-aadA1, aadA1*
S100	Urine	Surgery	AP/CAZ/CZ/S	+	1600, 1000	*dfrA1-aadA1, aadA1*
S8	Urine	Surgery	AP/CTX/CPD/CAZ/CRO/CIP/CZ/ATM	+	700	Hypothetical protein
S10	Urine	Emergency	AP/CTX/CAZ/CPD/CRO/CZ/SXT/S	+	1600	*dfrA1-aadA1*
S32	Stool	Internal	AP/CPD/CZ	+	1600	*dfrA1-aadA1*
S42	Respiratory secretions	Surgery	AP/CTX/CAZ/CPD/CRO/TN/GM/IMI/CZ/ S	+	1600	*dfrA1-aadA1*
S47	Respiratory secretions	Infectious	AP/CTX/CAZ/CPD/CRO/TN/GM/IMI/CZ/SXT/S	+	1600	*dfrA1-aadA1*
S60	Urine	Surgery ICU	CRO/CZ/SXT/S	+	1600	*dfrA1-aadA1*
S67	Wound	Surgery	AP/CTX/CRO/CIP/PTZ/IMI/ATM/MEM/SXT/S	+	1600	*dfrA1-aadA1*
S46	Respiratory secretions	NICU	AP/CZ/SXT/S	+	1700, 1600, 1000, 750	*dfrA12-orfF-aadA2, dfrA1-aadA1, aadA1, dfrA15-aadA1*
S92	Stool	Infectious	AP/TN/CZ/SXT/S	+	1700, 1600, 1000, 750	*dfrA12-orfF-aadA2, dfrA1-aadA1, aadA1, dfrA15-aadA1*
S54	Urine	Surgery	AP/CZ/SXT/SXT/S	+	1700, 1600, 1000, 600, 500	*dfrA12-orfF-aadA2, dfrA1-aadA1, aadA1*, Hypothetical protein
S88	Blood	Infectious	AP/TN/CZ/SXT/S	+	1700, 1600, 1000, 600, 500	*dfrA12-orfF-aadA2, dfrA1-aadA1, aadA1,* Hypothetical protein
S91	Blood	Surgery	AP/CTX/CPD/TN/CZ/SXT/S	+	1700, 1600, 1000, 600, 500	*dfrA12-orfF-aadA2, dfrA1-aadA1, aadA1,* Hypothetical protein
S73	Urine	Infectious	AP/CRO/CZ/SXT/S	+	1700, 1600	*dfrA12-orfF-aadA2, dfrA1-aadA1*
S85	Urine	Surgery	AP/CPD/TN/CZ/SXT	+	1700, 1600	*dfrA12-orfF-aadA2, dfrA1-aadA1*
S79	Stool	Surgery	AP/CPD/CZSXT/S/SXT	+	1700, 1600, 1000, 750, 700, 500	*dfrA12-orfF-aadA2, dfrA1-aadA1, aadA1, dfrA15-aadA1,* Hypothetical protein
S80	Urine	Surgery	AP/CPD/CAZ/CIP/CZ/SXT/S	+	1700, 1600, 1000, 750, 700, 500	*dfrA12-orfF-aadA2, dfrA1-aadA1, aadA1, dfrA15-aadA1,* Hypothetical protein
S89	Blood	Pediatric	AP/CPD/CZ/S	+	1700, 1600, 1000, 750, 700, 500	*dfrA12-orfF-aadA2, dfrA1-aadA1, aadA1, dfrA15-aadA1,* Hypothetical protein

SXT: Trimethoprim-sulfamethoxazole, CIP: Ciprofloxacin, TN: Tobramycin, GM: Gentamicin, CRO: Ceftriaxone, CTX: Cefotaxime, CAZ: Ceftazidime, ATM: Aztreonam, AP: Ampicilin, S: Streptomycin, CZ: Cefazolin, CPD: Cefpodoxime.

Gene cassettes of *dfrA1-aadA1, aadA1, dfrA12-orfF-aadA2* and *dfrA15-aadA2* were found in 26 (56.5%), 20 (43.5%), 10 (21.7%) and 7 (15.2%) isolates, respectively. The 1000 and 1600 bp fragments, which contained *dfrA1-aadA1, aadA1*, were the most frequents. The nucleotide sequences of gene cassettes reported in this study have been submitted to GenBank under accession numbers MF589545 for *aadA* and MF589546 and MF589547 for *dfrA*. The relationship between class I integron with resistance to 16 antibiotics was statistically analyzed (Table 3). Isolates contained class I integron showed significantly higher resistance to ciprofloxacin (p= 0.002), streptomycin (p=0.004) and Cotrimoxazole (p=0.041).

**Table 3.** Relatitionship of class I integron and antibiotic resistance among 46 MDR *Citrobacter* isolates.

**Antibiotics**	**Integron-positive isolates**			**Integron-negative isolates**			p value
	
	R (%)	S (%)	I (%)	R (%)	S (%)	I (%)	
Aztreonam	15 (32.6)	14 (30.4)	1 (2.2)	4 (8.7)	11 (23.9)	1 (2.2)	0.257
Imipenem	2 (4.3)	24 (52.2)	4 (8.7)	0 (0)	13 (28.3)	3 (6.5)	0.53
Cefazolin	26 (56.2)	0 (0)	4 (8.7)	16 (34.8)	0 (0)	0 (0)	0.126
Ceftazidime	14 (30.4)	12 (26)	4 (8.7)	8 (17.4)	8 (17.4)	0 (0)	0.302
Cefpodoxime	15 (32.6)	10 (21.7)	5 (10.9)	6 (13)	7 (15.2)	3 (6.5)	0.708
Tazobactam-Piperacillin	6 (13)	17 (36.9)	7 (15.2)	0 (0)	12 (26)	4 (8.7)	0.152
Gentamicin	6 (13)	24 (52.2)	0 (0)	2 (4.3)	14 (30.4)	0 (0)	0.523
Tobramycin	16 (34.8)	13 (28.3)	1 (2.2)	6 (13)	9 (19.5)	1 (2.2)	0.573
Meropenem	3 (6.5)	25 (54.3)	2 (4.3)	0 (0)	14 (30.4)	2 (4.3)	0.362
Ciprofloxacin	15 (32.6)	13 (28.3)	2 (4.3)	0 (0)	15 (32.6)	1 (2.2)	0.002*
Ertapenem	2 (4.3)	22 (47.8)	6 (13)	1 (2.2)	13 (28.3)	2 (4.3)	0.808
Cotrimoxazole	22 (47.8)	6 (13)	2 (4.3)	6 (13)	9 (19.5)	1 (2.2)	0.041*
Cefotaxime	18 (39.1)	9 (19.5)	3 (6.5)	6 (13)	9 (19.5)	1 (2.2)	0.221
Ampicillin	29 (63)	0 (0)	1 (2.2)	15 (32.6)	1 (2.2)	0 (0)	0.299
Ceftriaxone	18 (39.1)	10 (21.7)	2 (4.3)	4 (8.7)	10 (21.7)	2 (4.3)	0.077
Streptomycin	23 (50)	7 (15.2)	0 (0)	5 (10.9)	11 (23.9)	0 (0)	0.004*

## DISCUSSION

Recent research shows an increase of *Citrobacter* isolates among urinary tract infection agents with high antibiotic resistance in developed countries (12–14). Our results of antibiotic susceptibility testing showed the highest resistance of *Citrobacter* isolates to cefazolin and ampicillin and highest sensitivity to carbapenems, tazobactam and gentamicin. These findings are consistent with the results of previous studies (15–17). A high percentage of *Citrobacter* isolates in Kermanshah with multi-drug resistance indicates the dissemination of antibiotic resistance genes in this opportunistic pathogen (7). On the other hand, the accumulation of resistance genes within integrons contributes to the spread of MDR strains among *Enterobacteriaceae* isolates (18). Class I integron is widely distributed among multidrug resistance of *Enterobacteriaceae* isolates (19). Studies in Malaysia and Egypt have reported the rate of class I integron in isolates of *Citrobacter* and *Enterobacteriaceae* with 50% and 51%, respectively (5, 20). The above results are compatible with our results for the frequency of class I integron among *Citrobacter* isolates in Kermanshah.

Class I integron has been found to carry resistance to several antimicrobial agents in bacteria. For instance, cassettes for resistance to fluoroquinolones, β-lactams, aminoglycosides, trimethoprim and chloramphenicol have been identified (5). According to research data, most of the resistant genes for aminoglycosides (*aad, aac*) are transmitted by class I integrin (21). The results of our study also suggest a statistically significant association between the presence of class I integron and resistance to streptomycin. In our study, a significant correlation between the presence of class I integron and resistance to ciprofloxacin was also noted, which is consistent with other studies (21, 22). Although the mutation in topoisomerase genes is the main mechanism of resistance to fluoroquinolones, recently proteins have been identified encoded by integrons and carried on plasmids which increases the bacterial permeability for quinolones (23, 24).

Gene cassettes with different sizes carried by class I integron in *Citrobacter* isolates are consistent with the results of other studies on *Enterobacteriaceae* family (25, 26). Similar to other studies, our results indicate that integrons can carry several cassettes simultaneously and contribute to the emergence of MDR strains (27, 28). In our study, the phenotypic resistance to the certain antibiotics was observed in isolates carried the corresponding gene cassettes. For instance, there is a significant association between the presence of dehydrofolate reductase and aminoglycoside aden-yltransferase cassettes with phenotypic resistance to trimethoprim-sulfamethoxazole and streptomycin, respectively. This indicates the expression of integron genes and their role in the phenotype of bacteria.

Our results show eight different patterns of class I integron gene cassettes. The DNA analysis of gene cassettes indicates several antibiotic resistance gene cassettes. Two variants of aminoglycoside adenyl transfers (*aadA1/aadA2*) were detected which encode aminoglycoside 3′-9-adenylyltransferases and confer the resistance to streptomycin and spectinomycin (29). Sequence analysis also revealed three variants of *dfrA* (*dfrA1/dfrA12/dfrA15*), which encode the dihydrofolate reductase gene, confer resistance to trimethoprim (29). The horizontal transmission of resistance genes between bacteria can occur and expand the gene cassettes (30). As indicated by our results and also supported by other studies, the *dfr* cassette is mostly associated with the *aadA* gene cassette (30). These observations suggest this combination of gene cassettes can reflect their co-transmission and stable integration (31). In the present study, the *dfrA1-aadA1* gene cassettes showed a high prevalence in Class I integron, which is consistent with the results of other studies on *Enterobacteriaceae* isolates (32, 33). According to the previous research, Class I integron contained the *aadA1* or *dfrA1-aadA1* cassettes are commonly found in *E. coli* isolates in Europe (34–36). Similarly, these gene cassettes have been reported in Asian countries (37, 38). In some studies in Iran, the 5-arr, *aacA4-orfD, aadA5-dfrA17, dfrA1, aadA1-dfrA1* and *aadA2-dfrA12-orfF* cassettes have been reported as the most common cassettes in *Klebsiella pneumoniae* and *E. coli* isolates (29, 39–40). It seems that the *aadA* and *dfrA* gene cassettes in Iran are also prevalent

In conclusion, our results indicate a high prevalence of MDR among *Citrobacter* isolates in Kermanshah. A high frequency of class I integron and the associated gene cassettes, in particular *dfr* and *aadA*, present in MDR strains of *Citrobacter* isolated from hospitalized patients. They may play an important role in the creation and transmission of MDR strains. Statistical analysis indicates the association of integration class I and MDR isolates in this opportunistic pathogen, which needs continues surveillance in health care centers.
